# A prevalence of cardiometabolic risk factors among a rural Yoruba south-western Nigerian population: a population-based survey

**Published:** 2010-02

**Authors:** OO Oladapo, AO Falase, L Salako, O Sodiq, K Shoyinka, K Adedapo

**Affiliations:** Cardiovascular Unit, Department of Medicine, University College Hospital, College of Medicine, University of Ibadan, Nigeria; Cardiovascular Unit, Department of Medicine, University College Hospital, College of Medicine, University of Ibadan, Nigeria; Department of Pharmacology and Therapeutics, University of Ibadan, Nigeria; Country office, World Health Organisation, Lagos, Nigeria; Primary Health, Egbeda Local Government, Egbeda, Oyo State, Nigeria; Department of Chemical Pathology, College of Medicine, University of Ibadan, Nigeria

**Keywords:** cardiometabolic risk factors, cardiovascular disease, Nigeria

## Abstract

**Background:**

It has been hypothesised that rural sub-Saharan Africa is at an early stage of epidemiological transition from communicable to non-communicable diseases (NCD). Limited information exists about the prevalence of cardiometabolic risk factors and the burden of cardiovascular disease (CVD) in the adult Nigerian population, especially in the rural setting.

**Objectives:**

The aim of this study was to assess and describe the prevalence of several cardiometabolic risk factors in the sub-Saharan adult population of a rural Yoruba community, living in south-western Nigeria.

**Methods:**

The study was a descriptive, cross-sectional, random-sample survey. Participants were visited at home by trained nurses and community health extension workers (CHEW) who administered a questionnaire, took the relevant history, carried out clinical examinations and measurements and took samples for laboratory tests. They were supervised by primary healthcare physicians serving the community. The variables recorded comprised clinical history, CVD risk factors including blood pressure (BP), body mass index (BMI), waist circumference, blood sugar and serum lipid levels, cigarette use, and dietary habits. The participants included 2 000 healthy adults aged 18 to 64 years who had been living in the area for more than three years.

**Results:**

The average age was 42.1 ± 21.6, with 43.7% (873) being males and 56.3% (1127) females; 20.8% were hypertensive with BP ≥ 140/90 mmHg, 42.3% of the men and 36.8% of the women had BP ≥ 130/85 mmHg; 2.5% had diabetes, 1.9% had hypertriglycerideaemia, 43.1% had low HDL-C, 3.9% had general obesity, 14.7% had abdominal obesity, 3.2% were physically inactive, and 1.7% smoked cigarettes. Overall, 12.9% of the subjects were found to have at least one CVD risk factor. Using the Adult Treatment Panel (ATP) III criteria, 2.1% of men and 2.7% of women in the study population had at least three of the criteria, the commonest being HDL-C < 40 mg/dl in men or < 50 mg/dl in women, followed by BP ≥ 130/85 mmHg, then waist circumference > 88 cm in women or > 102 cm in men, followed by blood glucose ≥ 110 mg/dl.

**Conclusion:**

The results obtained from this study strongly suggest a high prevalence of cardiometabolic risk factors in this rural population and that the epidemiological transition is not restricted to the urban population. This serves as a wake-up call for action in the planning of health services for the management of CVD and other chronic NCDs.

## Summary

Worldwide, cardiovascular diseases (CVD) and the metabolic syndrome are major causes of morbidity and mortality, including sudden death.^1^-^3^ CVD is emerging as a significant health problem in sub-Saharan countries such as Nigeria, with a population of 140 million. These countries are undergoing epidemiological transition from communicable to non-communicable diseases (NCDs).^4^,^5^ Epidemiological transition has been closely linked to changes in the demographic, social and economic status of various populations, causing a global rise in chronic diseases, especially cardiovascular diseases (CVD).^4^,^5^

The prevalence of CVD, specifically stroke and heart attack is on an upward trend in the sub-Saharan region, accounting for one-tenth of all deaths.^6^ Nigeria has a double burden of communicable and non-communicable diseases. Maternal and childhood mortality, tuberculosis, malaria fever and HIV/AIDS are still the leading causes of death. However, increased urbanisation with rapid rural–urban migration, demographic, environmental, social, cultural and behavioural changes might have led to unhealthy lifestyles, with increased motorisation, decreased physical activity, poor dietary habits, and tobacco use and alcohol consumption. These lead to a greater incidence of atherosclerosis, hypertension, diabetes, hyperlipidaemia and obesity, which are risk factors for CVD.^7^ More than 80% of CVD and its risk factors are preventable with cost-effective measures but there is a dearth of population-based data on risk factors in Nigeria.

In Nigeria, hypertension is the most common non-communicable disease and it has emerged as the most important risk factor for CVD, affecting 15 to 30% of the population.^8^-^10^ The prevalence of other CVD risk factors is not as clearly defined. The attributable risk for hypertension tends to be greater in developing economies because the low rates of detection and treatment in such countries result in a proportionately higher rate of complications. A large number of hypertensive patients present for the first time with fatal and non-fatal cardiovascular events such as heart failure, coronary artery disease (CAD)^11^ and stroke. These complications can be lethal with a poor prognosis and reduced life expectancy,^12^,^13^ resulting in loss of man-hours, diminished work productivity, a social burden, and increased health expenditure. All these have a devastating impact on the family and national economy.

The absence of well-developed programmes for identification and comprehensive CVD risk assessment and management of high-risk individuals may be responsible for this.^14^ The economic impact on the patient and family is enormous as the cost of care is borne out of pocket and it affects people usually at the peak of their productive years.

A high prevalence of CAD risk factors such as hypercholesterolaemia has been reported in newly diagnosed hypertensives^15^,^16^ and in Nigerians belonging to a high socioeconomic, westernised class.^17^ Most of these were hospital-based studies. In this study we examined the prevalence of selected cardiometabolic risk factors in a representative sample of an adult population dwelling in rural Nigeria where information about CVD, the disease burden and risk factors is sparse.

## Methods

We conducted a cross sectional survey from December 2002 to November 2005 in a representative sample of 2 000 adults, aged 18 to 64 years who were permanent residents of the Egbeda local government area (ELGA), a rural community in south-western Nigeria, with a population of 128 000. Agricultural production and livestock breeding are the main economic activities of ELGA residents, whose average annual income is less than US $1 000. The study protocol was evaluated and approved by the Ethics of Human Research Committee of the State Ministry of Health. Individual consent was obtained verbally and where possible by written consent.

A systematic random sample of dwellings was selected from lists drawn up by field enumerators. Consecutive eligible adults were selected as the respondents. A maximum of three respondents were chosen per household. Community health extension workers (CHEW) collected the data for the study after being trained in basic interviewing techniques and standard methods of obtaining physical measurements. Instruments were adopted from the WHO STEPS survey, and adapted to the local settings.

During the home visits, the interviewers collected information on each subject via structured patient questionnaires and physical assessment. Information obtained in step 1 included demographic profile (age, gender), socioeconomic profile (educational and income level), self-reported cardiac risk factors (smoking, level of physical activity, salt intake, fruit and vegetable intake and family history), pre-existing cardiovascular conditions and complications (hypertension, diabetes mellitus, ischaemic heart disease, stroke, kidney disease).

Cigarette smoking was defined as smoking at least one cigarette per day for at least one year. Data were also collected on other forms of tobacco use such as chewing and snuffing. Physical activity was assessed using a questionnaire that asked participants about their work-related activities. Physical activity was considered to be engaging in sustained physical activity for at least 30 minutes on five or more days per week, whether in leisure time or integrated into their everyday life.

A clinic visit was subsequently set up for each respondent at the nearest primary healthcare centre (PHC) to enable him/her to have physical measurements taken, with further assessment of any incident cardiometabolic risk factors. In step 2, we assessed physical status such as anthropometrics, which was measured by body mass index (BMI), (kg/m^2^) and waist circumference (WC) (cm). Overweight was defined as a BMI ≥ 25.0 kg/m^2^ and increased CV risk as waist circumference ≥ 88 cm for women and ≥ 102 cm for men.

Blood pressure (BP) was measured by trained health workers according to the guidelines of the International Society of Hypertension (ISH)/World Health Organisation (WHO; 1999) and the seventh Joint National Committee on hypertension (JNC-7).^18^-^20^ Measurements were taken using a standard mercury sphygmomanometer with appropriate-sized cuff. Three BP measurements were taken using the subject’s right arm with him/her in the sitting position after five minutes of rest, allowing one minute between measurements. The mean of three measurements was used as the final value. Participants with an elevated BP measurement had their BP measured again after one to two weeks; the average BP on the second visit was used as the criterion for the diagnosis and control of hypertension. In addition, all treated hypertensive patients had their BP measured after one to two weeks.

Hypertension was defined as systolic blood pressure (SBP) of ≥ 140 mmHg, diastolic blood pressure (DBP) of ≥ 90 mmHg, or current treatment with antihypertensive drugs in subjects with a history of hypertension. Awareness of hypertension meant a previous diagnosis of hypertension or high blood pressure. Controlled hypertension was defined as treated hypertension with a SBP < 140 mmHg and a DBP < 90 mmHg at the second BP measurement.

Venous blood samples were obtained via the antecubital vein for biochemical assessment in step 3, including fasting serum total cholesterol, high-density lipoprotein cholesterol (HDL-C), low-density lipoprotein cholesterol (LDL-C), triglycerides and fasting blood glucose levels. Diabetes was diagnosed either by a history of previously known diabetes or a fasting plasma glucose of ≥ 126 mg/dl, and impaired fasting glucose was defined as fasting plasma glucose of 100 to 125 mg/dl.^21^ Hypercholesterolemia was defined as a total cholesterol level ≥ 200 mg/dl, HDL-C < 40 mg/dl for men or < 50 mg/dl for women, and/or triglyceride concentration ≥ 150 mg/dl. The metabolic syndrome was defined according to Adult Treatment Panel (ATP) III criteria.^22^

## Statistical analysis

The data obtained were analysed using SPSS version 13.0 software (SPSS Inc., Chicago, Illinois, USA). Descriptive analysis of the variables was performed to process the data as tables. Continuous variables were described by calculating the means and standard deviation (SD). Categorical variables were described using frequency tables.

## Results

Table 1 shows the characteristic features of the final study population, including BMI, SBP, DBP, and blood glucose and lipid profiles. Two thousand persons were analysed, with a mean age and SD of 42.1 ± 21.6. Of these, 43.7% (873) were males while 56.3% (1 127) were females. The mean values of other variables were as follows: BMI 22.8 ± 7.9 kg/m^2^ (men), 25.6 ± 11.3 kg/m^2^ (women), waist circumference (WC) 87.1 ± 20.2 cm (men) and 89.5 ± 18.4 cm (women), SBP 132.9 ± 35.1 mmHg, DBP 81.6 ± 22.3 mmHg, fasting blood glucose 115.8 ± 51.7 mg/dl, total cholesterol 162.1 ± 53.6 mg/dl, LDL-C 110.3 ± 43.8 mg/dl, HDL-C 42.3 ± 9.5 mg/dl and triglycerides 139.7 ± 62.2 mg/dl.

**Table 1:** Distribution Of Selected Cardiometabolic Risk Factors In The Study Population

*Variables*	*Mean*	*SD*
Age (years)	42.1	21.6
Body mass index (kg/m^2^)		
Men	22.8	7.9
Women	25.6	11.3
Waist circumference (cm)		
Men	87.1	20.2
Women	89.5	18.4
Systolic blood pressure (mmHg)	132.9	35.1
Diastolic blood pressure (mmHg)	81.6	22.3
Fasting blood glucose (mg/dl)	115.8	51.7
Total cholesterol (mg/dl)	162.1	53.6
Triglycerides (mg/dl)	139.7	62.2
HDL-C (mg/dl)	42.3	9.5
LDL-C (mg/dl)	110.3	110.3

Tables 2 and 3 show the prevalence of CVD risk factors according to gender and 10-year age-group distribution, respectively. The overall prevalence of hypertension in Egbeda residents was 20.8% (21.1% among men and 20.5% among women). Of these, only 14.2% (11.4% in men and 16.5% in women) were known hypertensives and only 18.6% of the known hypertensives were on any form of treatment. Although women had a higher rate of self-reported hypertension, the treatment rate was similar to that found in men. This may have been due to the fact that some of the women had a history of hypertension in pregnancy. The prevalence of metabolic disorders was as follows: diabetes 2.5%, hypertriglycerideaemia 1.9%, low HDL-C 43.1%, general obesity 3.9% and abdominal obesity 14.7%.

**Table 2:** Prevalence Of Selected Cardiometabolic Risk Factors In Males And Females In The Study Population

*Risk factor*	*Men (n = 873) %*	*Women (n =1127) %*	*Overall (n = 2000) %*
Blood pressure ≥ 130/85 mmHg*	(369) 42.3	(415) 36.8	(784) 39.2
Hypertension (≥ 140/90mmHg)	(184) 21.1	(231) 20.5	(415) 20.8
Newly diagnosed	(163) 88.6	(193) 83.5	(356) 85.8
Self reported	(21) 11.4	(38) 16.5	(59) 14.2
Receiving treatment past 3 months	(4) 19.0	(7) 18.4	(11) 18.6
Blood glucose ≥ 110 mg/dl*	(32) 3.7	(68) 6.0	(100) 5.0
Diabetes	(18) 2.1	(31) 2.8	(49) 2.5
Newly diagnosed	(7) 38.9	(6) 19.4	(13) 26.5
Self reported	(11) 61.1	(25) 80.6	(36) 73.5
Receiving treatment past 3 months	(5) 45.4	(8) 32.0	(13) 36.1
Total cholesterol ≥ 200 mg/dl	(25) 2.9	(38) 3.4	(63) 3.2
LDL-C ≥ 130 mg/dl	(9) 1.0	(8) 0.7	(17) 0.9
HDL-C < 40 mg/dl (men)* or < 50 mg/dl (women)*	(345) 39.5	(517) 45.9	(862) 43.1
Triglyceride ≥ 150 mg/dl*	(16) 1.8	(21) 1.9	(37) 1.9
BMI (kg/m^2^)			
< 18.5	(560) 64.1	(658) 58.4	(1218) 60.9
18.5–24.9	(283) 32.4	(421) 37.4	(704) 35.2
25–29.9	(l7) 1.9	(21) 1.8	(38) 1.9
≥ 30	(13) 1.5	(27) 2.4	(40) 2.0
Waist circumference			
Normal (< 80 cm women, < 94 cm men)	(762) 87.3	(944) 83.7	(1706) 86.5
Increased risk (80–88 cm women, 94–102 cm men)	(79) 9.0	(91) 8.1	(170) 8.5
Substantially increased risk (> 88 cm women, > 102 cm men)*	(23) 2.6	(101) 8.9	(124) 6.2
Tobacco use			
Current smokers	(33) 3.8	(0) 0.0	(33) 1.7
Current smokeless products	(0) 0.0	(12) 1.1	(12) 0.6
Family history of MI	(0) 0.0	(0) 0.0	(0) 0.0
Family history of stroke	(37) 4.2	(56) 5.0	(93) 4.7
Physical inactivity	(12) 1.4	(52) 4.6	(64) 3.2
Diet < 3 servings of fruit/vegetables daily	(785) 89.9	(872) 77.3	(1657) 82.9

*ATP III criteria

**Table 3:** Prevalence Of Selected Cardiometabolic Risk Factors By Age In The Study Population

	*Age (years)*					
	*18–24*	*25–34*	*35–44*	*45–54*	*55–64*	*Overall*
*Risk factors*	*(n) %*	*(n) %*	*(n) %*	*(n) %*	*(n) %*	*(n) %*
Hypertension	(3) 1.5	(39) 1.9	(155) 7.7	(171) 8.5	(47) 2.3	(415) 20.8
Diabetes mellitus	(1) 0.1	(4) 0.2	(29) 1.5	(12) 0.6	(3) 0.2	(49) 2.5
Hypertriglyceridaemia	(0) 0.0	(0) 0.0	(10) 0.5	(19) 1.0	(8) 0.4	(37) 1.9
Hypercholesterolaemia	(0) 0.0	(1) 0.1	(17) 0.9	(26) 1.3	(19) 1.0	(63) 3.2
Current smokers	(9) 0.5	(17) 0.9	(6) 0.3	(0) 0.0	(1) 0.1	(33) 1.7
Waist circumference						
> 80 cm (women)	(0) 0.0	(19) 0.9	(53) 2.7	(89) 4.4	(31) 1.6	(192) 9.6
> 94 cm (men)	(0) 0.0	(2) 0.1	(52) 2.6	(24) 1.2	(24) 1.2	(102) 5.1
Physical inactivity	(0) 0.0	(2) 0.1	(9) 0.4	(17) 0.8	(36) 1.8	(64) 3.2

In Tables 3, there was a clustering of risk factors, especially hypertension, diabetes, lipid abnormalities and abdominal obesity within the 35 to 44, and 45 to 54 years age groups of our sample. The prevalence of cigarette smoking was 1.7% and physical inactivity was 3.2%. Cigarette smoking and physical inactivity were found in the younger and older age groups, respectively. Smoking was the main form of tobacco use in Egbeda and this was found in men only, with a prevalence of less than 1.7%. Most smoked fewer than five cigarettes daily. Women used smokeless tobacco with a prevalence of 0.6%.

In this study, almost all responders admitted consumption of fruit on most days of the week and vegetables on a daily basis but this was found to be less than the recommended daily intake (82.9% consumed less than three helpings of fruit/vegetables daily). This was due mainly to affordability and the expression of fear of frequent bowel motions by some.

Table 4 shows the prevalence of multiple cardiometabolic risk factors by age in the study population. Overall, 12.9% of the subjects were found to have at least one CVD risk factor, 8.6% had two risk factors, 3.0% had three and 1.3% had four or more risk factors.

**Table 4:** Prevalence Of Multiple Cardiometabolic Risk Factors By Age In The Study Population

	*Age (years)*					
	*18–24*	*25–34*	*35–44*	*45–54*	*55–64*	*Overall*
*Risk factors*	*(n) %*	*(n) %*	*(n) %*	*(n) %*	*(n) %*	*(n) %*
2	(0) 0.0	(13) 0.7	(57) 2.9	(63) 3.2	(38) 1.9	(171) 8.6
3	(0) 0.0	(0) 0.0	(30) 1.5	(21) 1.0	(9) 0.4	(60) 3.0
≥ 4	(0) 0.0	(2) 0.1	(11) 0.5	(5) 0.3	(8) 0.4	(26) 1.3

Using the ATP III criteria, the prevalence of BP ≥ 130/85 mmHg was 39.2% [higher in men (42.3%) than in women (36.8%)], waist circumference > 88 cm in women and > 102 cm in men was 6.2%, blood glucose ≥ 110 mg/dl was 5.0%, HDL-C < 40 mg/dl in men and < 50 mg/dl in women was 43.1% and triglyceride ≥ 150 mg/dl was 1.9%. According to this guideline, 2.1% of men and 2.7% of women in the study population had at least three of the criteria, the commonest being HDL-C < 40 mg/dl in men or < 50 mg/dl in women, followed by BP ≥ 130/85 mmHg, then by waist circumference > 88 cm in women or > 102 cm in men, followed by blood glucose ≥ 110 mg/dl.

## Discussion

There is an emergence of chronic NCDs, including CVD in populations undergoing socioeconomic changes, and the rural sub-Saharan population is thought to be at an early stage of this epidemiological transition.^23^ CVD and its risk factors can adversely affect quality of life. The risk profile of a population can be utilised to predict the burden of atherosclerosis and its complications, such as stroke and CAD. The presence and number of CAD risk factors predict future cardiovascular events, especially heart attack and stroke in individuals with such risk factors.^24^

The present study was a population-based survey undertaken to determine the prevalence of cardiometabolic risk factors in a western Nigerian rural adult population, aged 18 to 64 years. The first major finding of our study revealed a relatively high frequency of hypertension (BP ≥ 140/90 mmHg) affecting 20.8% of the study population, low levels of HDL-C affecting 43.1%, and abdominal obesity affecting 14.7%. These three were the most common risk factors in the population. The pattern observed suggests that the health transition might be occurring at a rate faster than what should be seen in a population at an early stage of transition. This may worsen the rate of strokes and other cardiovascular events in the future.

Our study has contributed to determining the burden and distribution of cardiometabolic risk factors from age 18 to 64 years. Previous studies were done in elderly Nigerians over 55 years of age in the south-western portion of Nigeria,^25^ and were hospital based.

Using the ATP III criteria, we found the prevalence of cardiometabolic risk factors to be 2.1% in men and 2.7% in women. This rate was higher than that found in the city of Yaoundé in Cameroon and in three rural villages, using the same criteria, where the prevalence was < 0.5% in men and < 0.2% in women.^26^ A much higher rate of 24% in men and 32% in women was found in a nationwide study in the Seychelles.^27^ The prevalence of the metabolic syndrome in the Seychelles was noted to be similar to that in western countries.

The overall prevalence of hypertension in this study was 20.8%, an indication that hypertension affects a large proportion of the adult population in the rural community of Egbeda. A previous nationwide study^28^ and other studies^29^,^30^ found the prevalence rate ranging from 7% in the rural population to 20% in the urban population. This relatively higher prevalence rate of hypertension in a rural setting at an early stage of the economic and health transition is worrisome, since Egbeda is populated mainly by farmers, artisans and petty traders. The health systems serving the population are inadequately developed to tackle the burden of CVD. High and increasing rates of hypertension have been reported in rural communities in India^31^ and Indonesia.^32^

In Nigeria, services are offered free at primary healthcare centres (PHCs), but in spite of this, only a small proportion (18.6%) of patients with high blood pressure were on any form of treatment within three months prior to our study. Factors identified as contributory included care-seeker and caregiver apathy to chronic health issues which appeared to be non-incapacitating. The care seekers also complained of difficulties of transportation to clinics, missing the day’s work (most were daily-paid/self-employed workers who depended on the day’s earnings), competing issues, no drugs on arrival at PHCs, and no equipment or personnel in the facilities. Most of the PHCs had no programmes for chronic care in their practices. There was a dearth of personnel trained in long-term care, as well as inadequate medical records, medical supplies, equipment and drugs in almost all cases.

Our study is the first known documentation of the population prevalence of BP ≥ 130/85 mmHg, which was found in 39.2% of the study population. This risk factor will probably increase the burden of CVD in the near future if primordial and primary prevention are not instituted early enough. Therefore, there is an urgent need for early identification and treatment of persons in the pre-hypertensive state, using non-pharmacological methods of weight reduction, increased physical activity and increased consumption of fruit and vegetables, with a reduction in salt intake.

The results of our study showed that the prevalence of diabetes mellitus was 2.5% and this was similar to the national crude prevalence rate of 2.8%.^28^ The Egbeda residents with diabetes were more likely to be aware of their condition than the hypertensives (73.5% vs 14.2%, respectively). The treatment rate for diabetes was also higher than that for hypertension (36.1% vs 18.6%, respectively). It appeared that the reasons adduced for these included the fact that the presence of glucose in the urine in the study population prompted review for further management by a more senior member of the health team more often than detection of high blood pressure. We discovered that urine was tested in a few cases during antenatal care and in those who had observed the unusual presence of sugar ants around their latrines. Since urine testing is readily available and assessable in this community, the service can be up-scaled for early detection of diabetes.

The clustering of cardiometabolic risk factors in middle age is also worthy of note, indicating the need for a comprehensive and integrated approach to tackle CVD. It has been observed that the average human being is likely to experience a rise in body weight and blood pressure with age under the conditions of modern life.^23^ The REACH registry showed that most hypertensive patients have additional CVD risks.^33^ None of the participants had had an assessment of their lipid profiles prior to this study. We discovered that the PHCs had no facility for the testing of lipids; moreover, the health givers lacked the knowledge of the role of lipids in CVD. Low levels of HDL-C were found in 43.1% of the study population despite the relatively normal levels of total cholesterol and triglycerides. In south Asians 70.3% were found to have HDL-C lower than 40 mg/dl.^34^

Cigarette smoking was uncommon in the study population; however, the impact of smokeless tobacco use on CVD remains to be further elucidated, especially as this was found to be restricted to women. This was unlike the high prevalence of cigarette smoking in South African men, but the number of cigarettes smoked was equally low.^35^

Abdominal obesity was more common in our study population, with 8.5% having increased risk and 6.2% having substantially increased risk, compared to 6.4 and 1.6%, respectively, as recorded in a previous study in Nigeria.^36^ This may suggest an apparent upward trend in this risk factor within the past decade due to increased exposure to a westernised lifestyle. Previous studies have shown that waist circumference was positively correlated with blood pressure and fasting blood glucose and was significantly associated with higher risks of hypertension and diabetes in Nigerians, Jamaicans and African-Americans.^37^ Two per cent of our study population were obese with a BMI > 30 kg/m^2^, unlike a higher rate of 8% found in an inner-city urban community in Nigeria (the two studies reported that more women than men were obese).^38^ We recommend that abdominal obesity be used in defining CVD risk factors in this population, rather than overall obesity.

## Limitations of this research

We were able to study only one of the diverse ethnic groups dwelling in rural areas of Nigeria, and different regions of the country are known to be at different levels of acculturation to a western lifestyle. Insulin resistance and C-reactive protein were not estimated in this study in order to reduce costs; however these would not have affected the outcome of our study objectives.

## Conclusion

The relatively high rate of cardiometabolic risk factors in this rural population is an indication that the epidemic of CVD is looming large in Nigeria, a country whose health services are already overburdened by tuberculosis and the HIV/AIDS epidemic. Results of this study could serve as a basis for advocacy, with an urgent call for action for the development of national programmes that would improve the control and management of cardiometabolic risk factors and CVD, along with other NCDS. Primordial and primary population-based strategies, lifestyle modification, health education and health promotion would be cost effective in reducing CVD morbidity and mortality in this low-resource setting. Increased consumption of fruit and vegetables, use of appropriate cooking oil, and reduction of salt in food are good starting points for modifying some of these risk factors. Although the rate of cigarette smoking is low, smoking should be discouraged further.

## References

[1] Yusuf S, Reddy S, Ounpuu S, Anand S (2001). Global burden of cardiovascular diseases. Part 1; General considerations, the epidemiologic transition, risk factors, and impact of urbanization.. Circulation.

[2] Zimmet P, Magliano D, Matsuzawa Y, Albert G, Shaw J (2005). The metabolic syndrome: a global public health problem and a new definition.. J Atheroscler Thromb.

[3] Lakka H-M, Laaksonen DE, Lakka TA (2002). The metabolic syndrome and total and cardiovascular mortality in middle-aged men.. J Am Med Assoc.

[4] Omran AR (1974). The epidemiologic transition: A theory of the epidemiology of population change.. Milbank Mem Fund Q.

[5] Olshansky SJ, Ault AB (1986). The fourth stage of the epidemiologic transition: The age of delayed degenerative diseases.. Milbank Q.

[6] Akinboboye O, Idris O, Akinboboye O, Akinkugbe O (2003). Trends in coronary artery disease and associated risk factors in sub-Saharan Africans.. J Hum Hypertens.

[7] Stamler J, Dyer AR, Shekelle RB, Neaton J, Stamler R (1993). Relationship of baseline major risk factors to coronary and all-cause mortality, and to longevity: findings from long-term follow-up of Chicago cohorts.. Cardiology.

[8] Akinkugbe OO (1999). Current epidemiology of hypertension in Nigeria.. Arch Ibad Med.

[9] Akinkugbe OO (2000). Non-communicable disease, the next epidemic: Nigeria’s preparedness.. Nig J Clin Pract.

[10] Kadiri S (2005). Tracking cardiovascular disease in Africa.. Br Med J.

[11] Falase AO, Oladapo OO, Kanu EO (2001). Relatively low incidence of myocardial infarction in Nigerians.. Cardiologie Tropicale.

[12] Mckee PA, Castelli WP, McNamara PM, Kannel WB (1971). The Natural History of Congestive Heart Failure: the Framingham study. N Engl J Med.

[13] Howson CP, Reddy KS, Ryan TJ, Bale JR (1998). Control of Cardiovascular Disease In Developing Countries..

[14] Mendis S, Abegunde D, Oladapo O, Celluti F, Nordet P (2004). Barriers to management of cardiovascular risk in low-resource setting using hypertension as an entry point.. J Hypertens.

[15] Opadijo OG, Akande AA, Jimoh AK (2004). Prevalence of coronary heart disease risk factors in Nigerians with systemic hypertension.. Afr J Med med Sci.

[16] Taylor OG, Oyediran OA, Bamgboye AE, Afolabi BM, Osuntokun BO (1996). Profile of some risk factors for coronary heart disease in a developing country, Nigeria.. Afr J Med med Sci.

[17] Ogunowo PO, Ekpo EB, Odigwe CD, Andy JJ (1989). A clinical profile of patients with coronary artery disease in Nigeria.. Trop Geogr Med.

[18] (1999). 1999 WHO/ISH guidelines for the management of hypertension.. J Hypertens.

[19] Chobanian A, Bakris G, Black H, Cushman W, Green L, Izzo J (2003). Seventh report of the Joint National Committee on Prevention, Detection, Evaluation and Treatment of High Blood Pressure.. Hypertension.

[20] Chobanian AV, Bakris GL, Black HR, Cushman WC, Green LA, Izzo JL (2003). The seventh report of the Joint National Committee on Prevention, Detection, Evaluation, and Treatment of High Blood Pressure: the JNC 7 report.. J Am Med Assoc.

[21] (2004). Diagnosis and classification of diabetes mellitus.. Diabetes Care.

[22] (2001). Executive summary of the third report of the National Cholesterol Education Programme (NCEP) expert panel on detection, evaluation and treatment of high blood cholesterol in adults (Adult Treatment Panel III).. J Am Med Assoc.

[23] Forrester T, Cooper RS, Weatherall D (1998). Emergence of Western diseases in the tropical world: the experience with chronic cardiovascular diseases.. Br Med Bull.

[24] Pearson TA, Blair SN, Daniels SR (2002). AHA Guidelines for Primary Prevention of Cardiovascular Disease and Stroke: 2002 update: Consensus Panel Guide to Comprehensive Risk Reduction for Adult Patients without Coronary or Other Atherosclerotic Vascular Diseases.. Circulation.

[25] Ezenwaka CE, Akanji AO, Akanji BO, Unwin NC, Adejuwon CA (1997). The prevalence of insulin resistance and other cardiovascular disease risk factors in healthy elderly southwestern Nigerians.. Atherosclerosis.

[26] Fezeu L, Balkau B, Kengne AP, Sobngwi E, Mbanya JC (2007). Metabolic syndrome in a sub-Saharan African setting: central obesity may be the key determinant.. Atherosclerosis.

[27] Kelliny C, William J, Riesen W, Paccaud F, Bovet P (2008). Metabolic syndrome according to different definitions in a rapidly developing country of the African region.. Cardiovasc Diabetol.

[28] Akinkugbe OO (1997). Final report of a national survey on non-communicable diseases in Nigeria..

[29] Cooper R, Rotimi C, Ataman S, McGee D, Osotimehin B, Kadiri S (1997). The prevalence of hypertension in seven populations of West African origin.. Am J Publ Hlth.

[30] Kadiri S, Walker O, Salako BL, Akinkugbe O (1999). Blood pressure, hypertension ad correlates in urbanized workers in Ibadan, Nigeria: a revisit.. J Hum Hypertens.

[31] Singh RB, Sharma JP, Rstogi V, Niaz MA, Singh NK (1997). Prevalence and determinants of hypertension in the Indian social class and heart survey.. J Hum Hypertens.

[32] Ng N, Stenlund H, Bonita R, Hakimi M, Wall S, Weinehall L (2006). Preventable risk factors for noncommunicable diseases in rural Indonesia: prevalence study using WHO STEPS approach.. Bull Wld Hlth Org.

[33] Bhatt DL, Steg PG, Ohman EM, Hirsch AT, Ikeda Y, Mas JL, Goto S, Liau CS, Richard AJ, Rother J, Wilson PW (2006). REACH Registry. International prevalence, recognition, and treatment of cardiovascular risk factors in outpatients with atherothrombosis.. J Am Med Assoc.

[34] Ahmad U, Frossard PM (2006). Coronary heart disease in south Asia: need to redefine risk.. Int J Cardiol.

[35] Thorogood M, Connor M, Tollman S, Lewando Hundt G, Fowkes G, Marsh J (2007). A cross-sectional study of vascular risk factors in a rural South African population: data from the Southern African Stroke Prevention Initiative (SASPI).. BMC Publ Hlth.

[36] Okosun IS, Forrester TE, Rotimi CN, Osotimehin BO, Muna WF, Cooper RS (1999). Abdominal adiposity in six populations of West African descent: prevalence and population attributable fraction of hypertension.. Obes Res.

[37] Okosun IS, Cooper RS, Rotimi CN, Osotimehin B, Forrester T (1999). Association of waist circumference with risk of hypertension and type 2 diabetes in Nigerians, Jamaicans, and African-Americans.. Diabetes Care.

[38] Lawoyin TO, Asuzu MC, Kaufman J, Rotimi C, Owoaje E, Johnson L, Cooper R (2002). Prevalence of cardiovascular risk factors in an African, urban inner city community.. West Afr J Med.

